# Multivariate immune defences and fitness in the wild: complex but ecologically important associations among plasma antibodies, health and survival

**DOI:** 10.1098/rspb.2013.2931

**Published:** 2014-03-22

**Authors:** Daniel H. Nussey, Kathryn A. Watt, Abigail Clark, Jill G. Pilkington, Josephine M. Pemberton, Andrea L. Graham, Tom N. McNeilly

**Affiliations:** 1 Institute of Evolutionary Biology, University of Edinburgh, Edinburgh, UK; 2 Institute of Infection and Immunity Research, University of Edinburgh, Edinburgh, UK; 3 Centre for Immunity, Infection and Evolution, School of Biological Sciences, University of Edinburgh, Edinburgh, UK; 4 Department of Ecology and Evolutionary Biology, Princeton University, Princeton, NJ, USA; 5 Moredun Research Institute, Pentlands Science Park, Bush Loan, Midlothian, UK

**Keywords:** immunity, fitness, strongyle nematodes, anti-nuclear antibodies, Soay sheep, natural selection

## Abstract

Despite our rapidly advancing mechanistic understanding of vertebrate immunity under controlled laboratory conditions, the links between immunity, infection and fitness under natural conditions remain poorly understood. Antibodies are central to acquired immune responses, and antibody levels circulating *in vivo* reflect a composite of constitutive and induced functional variants of diverse specificities (e.g. binding antigens from prevalent parasites, self tissues or novel non-self sources). Here, we measured plasma concentrations of 11 different antibody types in adult females from an unmanaged population of Soay sheep on St Kilda. Correlations among antibody measures were generally positive but weak, and eight of the measures independently predicted body mass, strongyle parasite egg count or survival over the subsequent winter. These independent and, in some cases, antagonistic relationships point to important multivariate immunological heterogeneities affecting organismal health and fitness in natural systems. Notably, we identified a strong positive association between anti-nematode immunoglobulin (Ig) G antibodies in summer and subsequent over-winter survival, providing rare evidence for a fitness benefit of helminth-specific immunity under natural conditions. Our results highlight both the evolutionary and ecological importance and the complex nature of the immune phenotype in the wild.

## 1. Introduction

Understanding the causes and consequences of variation in host immune responses and susceptibility to infection is a central challenge in evolutionary ecology, biomedicine and epidemiology [1,2]. Studies linking immunity, parasite burdens and demographic rates in populations experiencing natural selection can provide crucial insight into both the selective forces maintaining immunogenetic variation and the consequences of that variation for host population dynamics [3–5]. Antibodies play a pivotal role in the vertebrate immune response; they are produced by B lymphocytes either spontaneously (so-called ‘natural’ antibodies) [6] or following stimulation with antigen, and exist as different isotypes with different functional properties [7]. Antibodies circulating in the blood of an individual at a given point in time will include a range of isotypes with different specificities (i.e. molecules bound) and affinities (i.e. bond strengths), reflecting that individual's genotype, environmental and antigenic experiences. While the importance of both natural and parasite-specific antibodies for protection from infection is increasingly well established in laboratory mice, domestic species and humans [7–10], the way in which they associate with health and fitness in natural populations has not been comprehensively examined [11,12]. Here, we examine the patterns of covariation among circulating antibody concentrations of different functional isotypes and specificities, and test their associations with fitness-related traits in immunologically experienced, free-living adult mammals.

There is mounting evidence that strong associations exist between measures of antibody-mediated immunity and fitness-related traits in natural populations [2]. The bulk of this evidence comes from studies of survival in juvenile animals [13], although there is also some support from immunologically mature and experienced adults [14–17]. The vast majority of these data come from wild birds, where the lack of immunological reagents and parasitological data has tended to limit researchers to using one or a few broad functional assays, typically associated with antibody activity against novel or recently inoculated, non-pathogenic antigens (e.g. sheep red blood cells [4,11]). This work has suggested important fitness benefits (e.g. improved health and survival) and fitness costs (e.g. reduced resources for growth or reproduction) associated with variation in immune phenotype in nature [2]. We do not currently know how well such broad immunological assays capture an individual's ability to mount an antibody response to parasitological challenge, however, nor do we understand the relative importance of cross-reactive antibodies capable of binding novel antigens versus induced antibody responses against prevalent parasites in natural settings [12,18]. Recent studies in birds and domestic ungulates that have measured a range of immunological traits suggest that simple overarching axes of variation are hard to identify and patterns of covariation among measures are often quite weak [13–16]. However, we have yet to take the crucial step of linking such a characterization of the multivariate immune phenotype to health and fitness measures in the wild.

In this study, we measured induced antibodies that bind antigens from ecologically important and highly prevalent parasites, and constitutively produced ‘natural’ antibodies that bind either novel non-self antigens or self antigens; we then assessed their associations with fitness-related measures in immunologically experienced adults in a wild Soay sheep population. There is a strong evidence that gastrointestinal nematode parasites are important selective agents and can influence the population dynamics of wild birds and mammals [17,19,20], including our study population [21,22]. Research in domesticated sheep points to an important role for antibodies that bind larval antigens—particularly those of isotypes immunoglobin (Ig) A and E, which have a central role in mucosal immunity—in the development on immunity to nematodes, with important implications for lamb growth and health [23,24]. Furthermore, there is growing appreciation of the immunological significance of diverse forms of ‘natural’ antibodies (NAbs), which are capable of binding antigens of which the individual has no prior experience [6]. NAbs are readily detected across vertebrate taxa and encompass low-affinity, polyreactive antibodies that will bind a variety of environmentally derived, non-self antigens, as well as germ-line encoded antibodies highly specific for self antigens associated with cellular damage [8,25]. Predominantly of IgM isotypes, but also including IgG and IgA, NAbs are thought to have important roles in defence against novel parasites or pathogens, immune regulation and clearance of damaged cells [8,25]. Recent studies in broiler hens and dairy cattle suggest NAbs against novel, non-self antigens (such as keyhole limpet haemocyanin or KLH) are both heritable and predictive of subsequent survival and reproductive performance traits [10,26,27]. However, little is currently known about the relationships between parasite-specific and self and non-self NAbs, and their associations with parasite defence and fitness, in natural populations.

We have previously used reagents developed by medical and veterinary immunologists to examine variation in T-cell phenotypes and both cross-reactive and parasite-specific antibody concentrations using samples collected as part of a long-term study of Soay sheep (*Ovis aries*) on St Kilda [28–30]. A previous study found negative associations between plasma concentrations of IgA binding larval antigens from a prevalent strongylid parasite, *Teladorsagia circumcincta*, and strongyle faecal egg counts (FECs) in Soay lambs, consistent with a role in resistance for these antibodies [28]. However, no such association was present in yearlings [28], and the relationship between such antibodies and parasite burdens and fitness in immunologically mature adults has not been investigated. More recently, we found that concentrations of antibodies against common nuclear constituents of mammalian epithelial cells (anti-nuclear antibodies, or ANAs) in plasma collected from adult females during summer positively predicted their survival of subsequent high-mortality winters [29]. ANAs at high levels have been associated with subsequent development of autoimmune disease in humans [31], but are likely to include self-antibodies directed at damage-associated molecular patterns (DAMPs), which may play an important role in immune-mediated homeostasis [25]. Based on weak (*r* < 0.3) but significant correlations between ANAs and both total IgG and pan-isotype antibodies against *T. circumcincta* in a small subset of the samples [29], and drawing upon evidence from human immunology and rheumatology [32], we hypothesized that the associations between ANAs and survival could reflect the fact that ANAs were closely associated with variation in an individual's overall antibody responsiveness [29].

Here, we aimed to examine the correlations among ANAs and 10 additional antibody variants—encompassing all four major mammalian isotypes and specificities for *T. circumcincta* and KLH antigens—and test their relationships with strongyle nematode FECs, body weight and over-winter survival in adult females captured across three summers that preceded high-mortality winters on St Kilda. Two recurrent challenges in correlational immunological field studies are, first, how well one or a few immune measures capture variation in the wider immune phenotype; and second, whether raised levels of an immune measure reflect recent patterns of exposure to infection or a protective immune response [4,16,33]. We explicitly tested whether close correlations existed among our 11 antibody measures and used principal component analysis to assess whether overall antibody variation could be meaningfully reduced to one or a few independent axes. The availability of immunological data, an indicator of parasite burden (FEC), and a key component of adult fitness (over-winter survival) provided a means to separate effects of exposure and resistance [4]. We predicted that, if antibody variation (captured as either individual variants or a principal component axis) reflected recent exposure to nematode infection, we should see positive correlations with FEC and negative relationships with weight and survival. Alternatively, if raised antibody levels reflected a protective response we would expect to see the opposite pattern.

## 2. Material and methods

### (a) Field and laboratory data collection

Soay sheep (*Ovis aries*) are descendants of domestic sheep that were present throughout northwest of Europe during the Bronze age, and probably reached the St Kilda archipelago 3000–4000 years ago. Since 1985, the individuals resident in the Village Bay area of Hirta have been the subject of long-term individual-based monitoring [34]. Individuals are caught and marked at birth in spring, and each August we re-capture as many sheep from the study population as possible using temporary traps. At capture, individuals are weighed, faecal sampled, and around 9 ml of whole blood is collected into a heparin tube and stored at 4°C. Within 48 h of collection, blood samples are centrifuged at 3000 r.p.m. (approx. 1500*g*) for 10 min, and the plasma is removed and stored at −20°C. Nematode eggs in faecal samples are counted shortly after collection, and strongyle FEC is estimated as the number of eggs per gram using a modified McMaster technique (following [21]; see [34] for further details of the population's history and fieldwork). On St Kilda, five nematode species contribute to this count, the most abundant being *T. circumcincta*, *Trichostrongylus axei* and *Trichostrongylus vitrinus* [35]. The vast majority of mortality occurs over winter (December–March), and the population dynamic is characterized by periods of low and rising sheep numbers followed by dramatic over-winter ‘crashes’ in which more than 50% of the population can die [34]. During these high-mortality ‘crash’ winters, regular mortality searches are conducted. The vast majority of carcasses are found and migration out of the study area is rare, so mortality can be determined with a high degree of confidence.

We used information collected on adult females (aged 2 years or more) caught in the Augusts preceding the high-mortality ‘crash’ winters of 1998/1999, 2001/2002 and 2004/2005. All animals were of known age, because they had been caught at birth. Previously, plasma samples from all individuals caught in these three summers had been assayed for ANAs [29]. We included these data in our analyses. We optimized sheep antibody assays to quantify concentrations of total IgA, IgM and IgG, anti-KLH IgA, IgM and IgG, and anti-*T. circumcinta* third larval stage (L3) IgA, IgM, IgG and IgE. Having established appropriate concentrations for our capture and detection antibodies, we conducted optimization assays to determine appropriate dilutions for our plasma samples for each assay type. We performed doubling dilutions on a set of test samples from 1 : 50 until ODs reached background levels, and determined the range of dilutions across which ODs decreased linearly for each assay. We then selected an appropriate dilution from the upper part of this linear range, and tested the repeatability of each assay by repeating the assay three to four times across 40–90 different plasma samples obtained from domestic sheep (kindly provided by Dr J. Houdijk at the SRUC). If sample repeatability (calculated following [36]) was 0.80 or higher we accepted the protocol, if not we re-optimized until this criterion was met. Full details of the optimized protocols for measuring all 11 antibodies are available in the electronic supplementary material, Methods file.

### (b) Data analysis

We obtained 239 samples from adult females for which we had a full set of 11 antibody measures across the three pre-crash August field seasons in 1998 (*n* = 83), 2001 (*n* = 75) and 2004 (*n* = 81). Two of these animals were not weighed at capture, and 12 were not faecal sampled and so did not have FEC data, but for all animals we could determine whether or not they had survived the subsequent crash winter using mortality and census records. Crash survival rates were 67% overall. The 239 samples came from 190 different females, seven of which were sampled in all 3 years and 35 of which were sampled in 2 of the years, leaving 148 females sampled in only 1 year. Since only three of the females caught across our 3 sampling years were older than 11 years, we incorporated this record within a final ‘11+ years old’ age group.

We examined the distributions of the antibody measures and, as they tended to approximate normality, elected not to transform them prior to further analyses. We estimated Pearson's correlation coefficients among all pairs of antibody measures. We also estimated variance inflation factors for each antibody measure to test whether levels of colinearity were present that might cause problems in subsequent linear models [37]. Variance inflation factors among measures were all below 3 (see electronic supplementary material, table S2), suggesting no major issue with colinearity [37]. To test whether variation among our 11 antibody measures could be reduced to one or a few independent axes, we ran a principal component analysis (PCA) including all 11 variables. The first axis produced by the PCA explained a moderate amount of overall variation (31%) and showed loadings consistent with an indicator of general antibody responsiveness (see Results). Other axes explained considerably less variance and were not easy to interpret biologically, so we elected to derive individual scores for the first axis only. We tested whether this axis predicted August FEC, weight and over-winter survival by including it in base models described below and testing significance with likelihood ratio tests (LRTs). We also compared the explanatory power of models including the first axis score (our putative ‘general antibody responsiveness’ measure) with our final models including significant separate antibody variables using AIC (see below for details of how these were determined), to test whether the first PCA axis explained as much variation as significant antibody measures fitted separately.

We proceeded to construct generalized linear mixed-effects models (GLMMs) to test for associations between the antibody measures and August FEC, August weight and survival of the subsequent ‘crash’ winter. All models were run in R, using the package ‘lme4’. All models included a random effect for individual to account for repeated measures of some individuals across the 3 sampling years. All models also included year of sampling and age as fixed factors to account for well-documented annual environment and age-related variation in FEC, weight and survival [34]. August weight was normally distributed and modelled using a Gaussian error structure. FEC values were grouped into multiples of 100 (i.e. 0, 1–100, 101–200, etc.) with the four observations with values greater than 600 grouped together at a value of 700 eggs g^−1^ to better approximate a Poisson distribution. However, the data remained over-dispersed, and to account for this we fitted a Poisson error and a log link function, while also including a random effect for observation to explicitly model the over-dispersion (following [38]). Over-winter survival was binary (0 = died, 1 = survived) and modelled using a binomial error and a logit link function. We included August weight in the survival GLMM, to account for the significant positive association that existed (addition of weight to GLMM of over-winter survival without antibody terms: 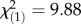, *p* < 0.001), but did not include FEC as it was not a significant predictor of survival (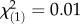, *p* = 0.93). All antibody variables were mean centred and unit s.d. standardized prior to inclusion in GLMMs to put them all on the same scale.

We began by determining for each antibody measure, first, whether it significantly predicted FEC, weight or survival when fitted in a GLMM without the inclusion of other antibody terms, and second, whether any relationship was linear or quadratic, as studies of wild animals have found curvilinear relationships between antibody levels and fitness-related traits which could reflect costs of immunity [39]. For each measure, both a linear and quadratic term were added to the relevant GLMM and their significance assessed based on LRTs. Where quadratic terms were significant, they were included in subsequent models, but if not they were dropped and only the linear terms were incorporated in subsequent analyses. We then tested for independent associations between antibody measures and our three response variables by including all antibody terms in initial GLMMs of weight, FEC and survival, and using both stepwise model simplification and model averaging approaches. In the stepwise models, all 11 antibody terms were included in a maximal GLMM, along with any quadratic terms previously identified as significant. The antibody term with the lowest *z*-value (the ratio of the estimated slope to its standard error) was dropped from the model, and a lack of significant change in model explanatory power was confirmed using a log-LRT. This proceeded sequentially, with the proviso that linear terms could not be dropped before their quadratic term, until all antibody terms remaining produced significant (*p* < 0.05) changes in model likelihood when dropped. To confirm that this was a reasonable final model, all dropped terms were sequentially reintroduced into this final model and a lack of significant change in explanatory power was confirmed by LRTs. The model averaging approach involved starting with the same maximal GLMM as above and running all possible models with different combinations of antibody terms, while retaining all non-antibody terms in all models, using the R package ‘MuMiN’. We generated model-averaged estimates for each antibody term from a top model set consisting of all models within four AIC units of the model with the lowest AIC [40].

## 3. Results

### (a) Correlations among antibodies and principal components analysis

The vast majority of the correlations among the antibody measures were positive (52 of 55; electronic supplementary material, table S1). The strongest correlations were within IgA and IgM isotypes, and between total IgG and anti-KLH IgG (see electronic supplementary material, table S1), while anti-Tc IgG were weakly associated with other assays for that isotype, and ANAs and anti-Tc IgE were generally weakly associated with other measures (see electronic supplementary material, table S1). PCA identified a first axis on which all antibody measures loaded in the same direction (explaining 31% of the variance), although some loadings were quite weak (see the electronic supplementary material, table S2). Subsequent PC axes explained considerably less variance and were not easy to interpret biologically. We therefore elected to derive individual scores for the first axis only, as a putative indicator of general antibody responsiveness, and compared the explanatory power of the scores with those of individual antibody measures in the GLMMs of weight, FEC and survival discussed below. Overall, this did not support the prediction that overarching variation in ‘general antibody responsiveness’ would correlate most strongly with traits like FEC, weight and survival. The first principal axis score did not significantly predict August FEC (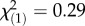, *p* = 0.59), nor over-winter survival (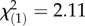, *p* = 0.15), and both final GLMMs including individual antibody terms (see the electronic supplementary material, tables S4 and S6, respectively) outperformed a model including the PCA score (ΔAIC = 12.12 and 11.87, respectively). First axis scores did significantly and positively predict August weight (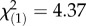, *p* = 0.04; *b* = 0.19 ± 0.09 SE), suggesting individuals with higher overall antibody concentrations were heavier. However, the final model of weight (see the electronic supplementary material, table S5) containing four different antibody terms explained far more variation in weight than the principal axis score alone did (ΔAIC = 24.70).

### (b) GLMMs of August faecal egg counts

**Figure 1.**
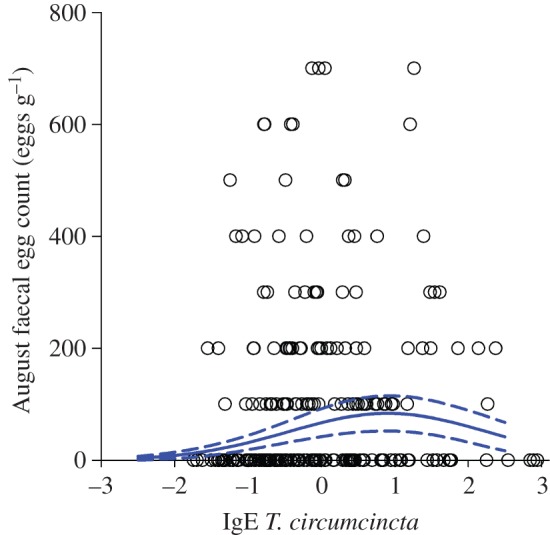
Scatter plots of raw data and GLMM predictions showing significant relationships between August strongyle FECs and an anti-*T. circumcincta* IgE antibody measure. The predicted relationship is based on the final stepwise model in the electronic supplementary material, table S4 estimated for females aged 7 years in 1998. (Online version in colour.)

There was a highly significant quadratic association between anti-Tc IgE concentration and August FEC (figure 1; electronic supplementary material, table S3). Stepwise simplification and model-averaging approaches provided consistent evidence that linear and quadratic anti-Tc IgE terms were highly significant independent of all other antibody measures (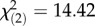, *p* < 0.001; electronic supplementary material, table S4). GLMMs predicted an increase in FEC with anti-Tc IgE up to around 1 s.d. above the mean for this antibody, followed by a plateau and possible decline (figure 1). Separating the data either side of this point revealed that while there was a significant increase in FEC with IgE up to 1 s.d. above the mean (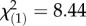, *p* < 0.01, *b* = 0.55 ± 0.20 s.e.), there was no significant decline in FEC beyond this point (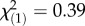, *p* = 0.53, *b* = −0.40 ± 0.65 s.e.).

### (c) GLMMs of August weight

**Figure 2.**
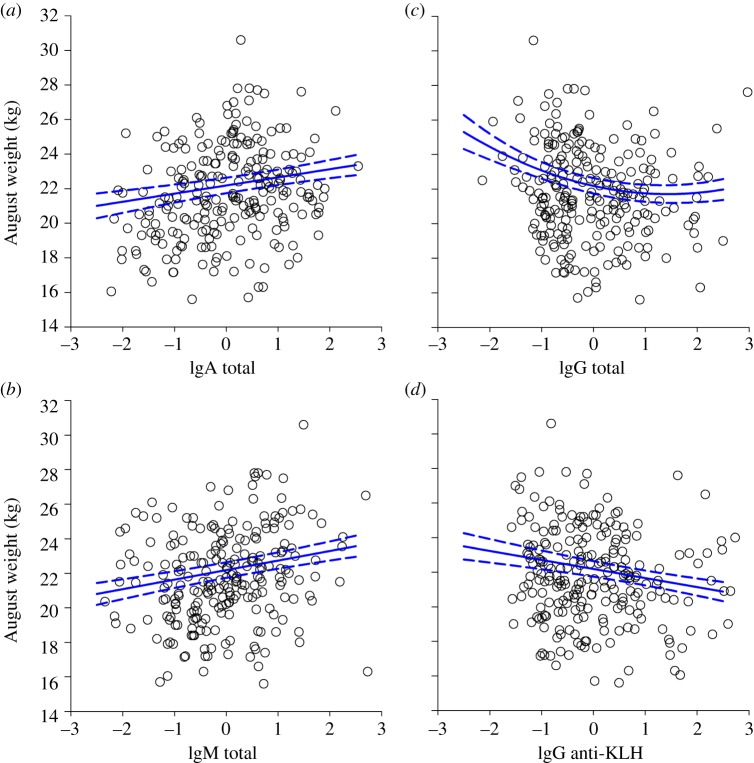
Scatter plots of raw data and GLMM predictions (see the electronic supplementary material, table S5) showing significant relationships between August weight and antibody measures: (*a*) total IgA, (*b*) total IgM, (*c*) total IgG and (*d*) anti-KLH IgG. The predicted relationship with weight is based on the final stepwise model in the electronic supplementary material, table S5 estimated for females aged 6 years in 1998. (Online version in colour.)

August weight was independently positively associated with total IgA and total IgM, and negatively with total IgG and anti-KLH IgG (figure 2; electronic supplementary material, table S5). Stepwise simplification modelling suggested that August weight increased linearly with total IgA (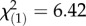, *p* = 0.01; figure 2*a*), and total IgM (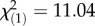, *p* < 0.001; figure 2*b*), but declined in a curvilinear fashion with total IgG (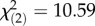, *p* < 0.01; figure 2*c*) and linearly with anti-KLH IgG (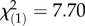, *p* < 0.01; figure 2*d*). Full details are provided in the electronic supplementary material, table S5, but we note that the model-averaging approach provided only marginal support for a significant effect of total IgA (*z* = 1.72, *p* = 0.09) and should thus be interpreted with caution, although the other three terms were highly significant using both approaches (*p* < 0.01).

### (d) GLMMs of over-winter survival

**Figure 3.**
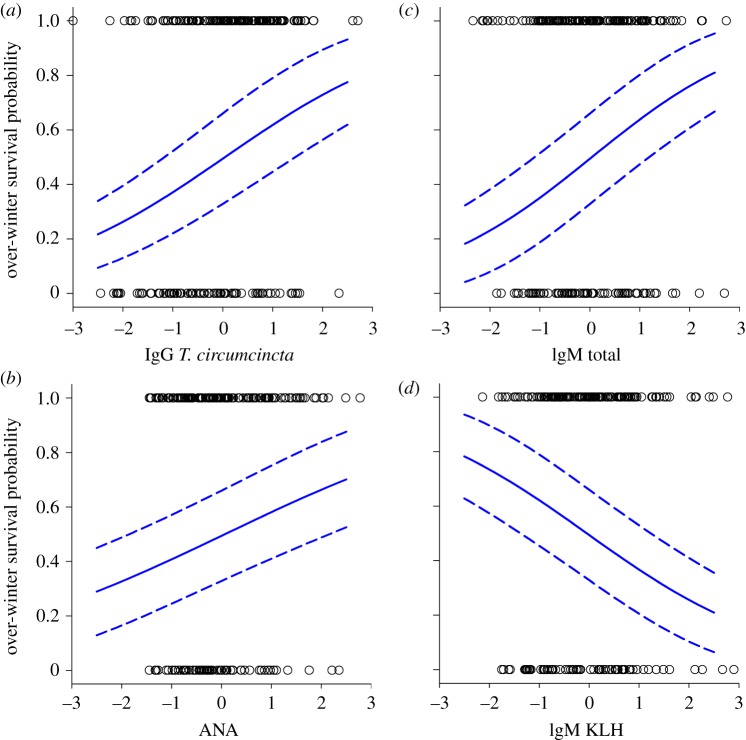
Scatter plots and GLMM predictions showing significant relationships between over-winter survival probability and antibody measures: (*a*) anti-*T. circumcincta* IgG, (*b*) ANAs, (*c*) total IgM and (*d*) anti-KLH IgM. The predicted relationship is based on the final stepwise model in the electronic supplementary material, table S6 estimated for females of mean overall weight aged 7 years in 1998. (Online version in colour.)

Females with higher concentrations of ANAs, anti-Tc IgG and Total IgM were more likely to survive the following winter (figure 3; electronic supplementary material, table S6). Independent of weight, age and year, total IgM, anti-Tc IgG and ANAs were significant predictors of survival following stepwise simplification (anti-Tc IgG: 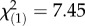, *p* < 0.01; figure 3*a*; ANA: 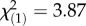, *p* = 0.05; figure 3*b*; total IgM: 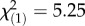, *p* = 0.02; figure 3*c*; see the electronic supplementary material, table S6 for full details). The model-averaging approach yielded very similar results, although ANA was marginally non-significant (*z* = 1.88, *p* = 0.06; electronic supplementary material, table S6). Interestingly, stepwise modelling identified an independent association between anti-KLH IgM and survival (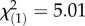, *p* = 0.03; figure 3*d*; electronic supplementary material, table S6), which was also marginally non-significant according to the model-averaging approach (*z* = 1.95, *p* = 0.05). There was no evidence that anti-KLH IgM had a significant relationship with survival when fitted on its own in the model (see electronic supplementary material, table S3), and further investigation revealed that its significance when other antibody measures were present in the model (see electronic supplementary material, table S6) was dependent on total IgM being included in the model. Removal of total IgM from the final model resulted in the anti-KLH IgM effect weakening substantially and becoming non-significant (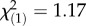, *p* = 0.28, *b* = −0.21 ± 0.19 s.e.). However, if we took the residuals from a regression of total IgM on anti-KLH IgM—which reflect variation in anti-KLH IgM that was independent of its positive correlation with total IgM—and fitted them to a model of survival excluding other antibody terms, their effect was significant and negative (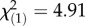, *p* < 0.05, *b* = −0.52 ± 0.24 s.e.). Thus, although total and anti-KLH IgM are positively correlated (*r* = 0.65), our models suggest that while a general survival benefit is associated with high total IgM, this benefit is likely to be cancelled out, to some degree, if an individual has high anti-KLH IgM relative to their total IgM concentrations.

## 4. Discussion

Our results fit well with emerging research documenting complex environment- or species-specific patterns of associations among immunological measures in veterinary and ecological studies [10,13–16]. For instance, we have demonstrated that, although correlations among different antibody variants are generally positive in direction, they are typically weak (over 90% of correlations less than 0.5). There was at best weak evidence for a single overarching axis of variation associated with general antibody responsiveness, with our first PCA axis explaining only 31% of overall variation, and we found no evidence that such an axis predicted variation in either our index of parasite burden (FEC) or subsequent survival. There was also no support for our previous hypothesis that plasma ANA concentrations might reflect overall antibody responsiveness [29], as ANA concentrations were only weakly associated with other antibody measures and their association with survival was independent of other measures.

No single antibody measure correlated positively with FEC and negatively with weight and survival (as predicted if variation in exposure was solely responsible for variation in antibody titers), nor *vice versa* (as predicted if variation solely reflected a protective response to infection). The complex pattern of relationships observed suggests that associations between antibody measures and with health and fitness proxies depend on the antibody specificity and isotype. Anti-Tc IgE increased with FEC initially and then plateaued, which could reflect differences in exposure to strongyle parasites (see below). Weight was positively related to total IgM and IgA levels, which might reflect variation in underlying condition influencing both total antibody levels and weight. However, to our surprise, weight was also strongly and negatively predicted by anti-KLH IgG, a result mirrored to some degree by the findings that individuals with low anti-KLH IgM relative to their total IgM levels had improved survival prospects. We discuss some possible explanations for these unanticipated negative relationships between antibodies and novel, non-self antigens and fitness-related traits below. Importantly, we identified an independent positive association between anti-Tc IgG concentrations and over-winter survival that cannot be readily explained in terms of either variation in exposure or condition as it is independent of the effects of either weight or FEC on survival. Although our data are correlational and the mechanisms responsible for associations between anti-Tc antibody levels and survival remain undetermined, this result constitutes—to our knowledge—the best currently available evidence for a survival benefit of helminth-specific immunity from a natural population.

### (a) Anti-*Teladorsagia circumcincta* antibodies, parasite burdens and survival

Veterinary studies of domestic sheep suggest that both IgA and IgE responses are critical to the development of immunity to *T. circumcincta* and other gastrointestinal nematodes in early life [23,41]. Specifically, it is proposed that an IgA response develops first, associated with slowed development of fourth-stage larvae and reduced parasite fecundity, and that a hypersensitivity response involving IgE develops later, directed at the incoming third-stage larvae, which is important in preventing their establishment in the gut [23,24,41]. Experimental and correlational studies of the Soay sheep population on St Kilda indicate that infection with *T. circumcincta* and other nematode parasites plays a role in the over-winter mortality of both lambs and adults [22,35,42]. A previous study on St Kilda found a significant negative correlation between plasma anti-Tc IgA and FEC in lambs at around four months of age [28]. However, in samples taken from adult females, we found little evidence for negative associations between IgE or IgA and FEC, nor of any further associations between these measures and subsequent survival. In fact, we found that FECs increased from low to moderate anti-Tc IgE before reaching a plateau (figure 1). This seems most likely to reflect heterogeneity in recent exposure and stage of infection, given that IgE responses appear generally short-lived, if they are detected at all, in adult sheep following experimental infection with larvae [43,44]. Although IgE and IgA against *T. circumcincta* may be important indicators of resistance in young animals that are in the process of developing immunity, recent studies suggest the immune response in chronically exposed, immunologically mature adults could be more geared towards damage limitation and tolerance of parasites [45,46].

The presence of a strong, positive association between anti-Tc IgG titres and over-winter survival suggests that there are important fitness consequences of variation in strongyle-specific immunity in adult Soay sheep. However, the considerable time lag between sampling in summer and the period of nutritional, parasitological and thermoregulatory challenge leading up to the winter mortality period (December–March) suggests anti-Tc IgG titres reflect some evolutionary and ecologically important, temporally stable aspect of immunity to these parasites. Although IgA and IgE isotypes have much shorter half-lives than IgG and may circulate at high levels only during acute infections, a previous study showed a pan-isotype measure of anti-Tc antibodies (which should be predominantly IgG) to be highly repeatable among individuals across years [47]. There is also evidence from domestic sheep for considerable antibody cross-reactivity among strongylid species, including the range of species that infect the Soay sheep [43,48]. It is therefore plausible that variation in IgG against *T. circumcincta* could reflect some temporally stable and repeatable aspect of an individual's immune response to strongyle parasites, which may play an important role in helping the individual to maintain homeostasis in the face of the interacting pressures of parasite damage and nutritional deprivation across the winter period. Further research into the precise strongyle antigens bound by antibodies in Soay sheep, their cross-reactivity among parasite species and life stages, as well the interaction between antibody titres and nutritional state is now required to test this hypothesis.

### (b) Natural antibodies, health and survival

Independent of associations with anti-strongyle antibody concentrations, total and natural antibodies of different isotypes were found to be important predictors of August weight and survival in adult female Soay sheep. Our models revealed that, independent of age or year, the heaviest females in August had higher total IgM and IgA, but lower total IgG and anti-KLH IgG concentrations (figure 2). Furthermore, independent of weight, age, year and the aforementioned association with anti-Tc IgG, females with high ANA and high total IgM accompanied by relatively low anti-KLH IgM were more likely to survive the subsequent winter (figure 3). Although the mechanisms behind this pattern of associations remain to be determined, it is clear that total IgM and antibodies binding ANAs and KLH are only weakly correlated, and are associated with weight and survival in complex, antagonistic ways in our study population.

There is growing evidence that germ-line encoded auto-antibodies that bind oxidation-specific epitopes and other DAMPs could perform an important role in identifying damaged cells for destruction and removal by macrophages [25,49]. It is possible that our ANA assay is in part measuring DAMP-binding antibodies and the maintenance functions of that class of natural antibody may underlie ANAs' association with over-winter survival and longevity in female Soay sheep [29]. Conversely, our results suggest that antibodies binding a novel, exogenous antigen (KLH) were negatively correlated with fitness-related traits. This is surprising given mounting evidence from livestock that antibodies against KLH are both heritable and positively associated with important production traits [10,26,27]. Differences in both environmental pressures and past selection history could explain these apparent differences, and it is conceivable that under the continuous parasite challenge and food limitation experienced by our study population—especially in the years studied here—high concentrations of anti-KLH antibodies may either come at a cost to more specific immune responses or reflect some form of immunopathology. Interestingly, some vaccination studies have suggested that high natural antibody concentrations can stymie the development of specific antibody responses to challenge [50]. Further investigation of the exact nature of the antigens bound by sheep antibodies in both ANA and KLH assays is important to better understand the mechanisms involved. However, whatever the underlying mechanisms, our data suggest that the relative production of natural self-reactive versus non-self antibodies may have important and potentially antagonistic fitness consequences, which could constrain selection on immunity.

## 5. Conclusion

In this study, we have documented strong and independent associations between plasma antibodies and fitness-correlated traits in a wild mammal. Determining whether raised antibody levels reflect differences in exposure to parasites or raised investment in protective immunity is challenging in correlative studies like ours. Through combined study of immune phenotype, indices of parasite burden and fitness, we would argue that we have been able to provide some evidence for fitness benefits of raised levels of both parasite-specific and natural antibodies in a wild population, which cannot readily be explained by variation in exposure to parasites. However, we have only explored antibody concentrations in one demographic group experiencing a particular phase of our study population's dynamic, in which both sheep density and parasite exposure were likely to be particularly high. It remains to be seen whether similar patterns will be observed in other demographic groups or different environmental conditions. Furthermore, we have focused on survival, which is only one aspect of lifetime fitness. Indeed, our previous work on ANAs revealed negative associations with fecundity [29], consistent with a reproductive cost of elevated concentrations of this antibody type. It will be interesting and important to determine whether anti-strongyle IgG, which have a stronger and more statistically robust association with survival than ANAs, are also negatively correlated with reproductive performance traits. Furthermore, having established that variation in single antibody measures can be highly heritable in this population [29,47], larger-scale, multivariate quantitative genetic studies are now required to determine to what extent circulating antibody measures are genetically correlated and the degree to which such correlations may constrain natural selection on the immune phenotype. Overall, our results strongly support the idea that only by considering the immune system as a complex, multivariate defence system in the context of relevant infectious agents will we be able to gain a meaningful understanding of the evolutionary and ecological causes and consequences of variation in immunological phenotypes in natural populations.

## Data accessibility

The dataset used in the analyses presented here is available on Dryad (10.5061/dryad.74k3m), with a 12-month embargo period in place. The data are also available now from the corresponding author on request, and we would strongly encourage anyone interested in using these data to contact the corresponding author.
